# Continuous Viewpoint Planning in Conjunction with Dynamic Exploration for Active Object Recognition

**DOI:** 10.3390/e23121702

**Published:** 2021-12-20

**Authors:** Haibo Sun, Feng Zhu, Yanzi Kong, Jianyu Wang, Pengfei Zhao

**Affiliations:** 1 Faculty of Robot Science and Engineering, Northeastern University, Shenyang 110169, China; sunhaibo@sia.cn (H.S.); wangjianyu@sia.cn (J.W.); 2 Shenyang Institute of Automation, Chinese Academy of Sciences, Shenyang 110016, China; kongyanzi@sia.cn (Y.K.); zhaopengfei@sia.cn (P.Z.); 3 Key Laboratory of Opto-Electronic Information Processing, Chinese Academy of Sciences, Shenyang 110016, China; 4 Institutes for Robotics and Intelligent Manufacturing, Chinese Academy of Sciences, Shenyang 110169, China; 5 University of Chinese Academy of Sciences, Beijing 100049, China

**Keywords:** active object recognition, continuous viewpoint planning, adaptive entropy regularization, dynamic exploration, proximal policy optimization

## Abstract

Active object recognition (AOR) aims at collecting additional information to improve recognition performance by purposefully adjusting the viewpoint of an agent. How to determine the next best viewpoint of the agent, i.e., viewpoint planning (VP), is a research focus. Most existing VP methods perform viewpoint exploration in the discrete viewpoint space, which have to sample viewpoint space and may bring in significant quantization error. To address this challenge, a continuous VP approach for AOR based on reinforcement learning is proposed. Specifically, we use two separate neural networks to model the VP policy as a parameterized Gaussian distribution and resort the proximal policy optimization framework to learn the policy. Furthermore, an adaptive entropy regularization based dynamic exploration scheme is presented to automatically adjust the viewpoint exploration ability in the learning process. To the end, experimental results on the public dataset GERMS well demonstrate the superiority of our proposed VP method.

## 1. Introduction

Visual object recognition plays an important role in the fields of computer vision and robotics. It has been successfully applied into a large number of tasks, e.g., autonomous driving, manipulation and grasping, monitoring security, transportation surveillance [1], etc.

Most recognition systems exclusively focus on static image recognition, that is, the systems take a single snapshot as input and generate a category label estimate as output [2]. It is easy to produce recognition errors when the single-view image can not provide enough information. However, the vision behavior of people is exploratory, probing, and searching in order to better understand their surroundings. For example, you will go to the front of a person to confirm when you can not identify him from his back. Thus, if the viewpoint of an agent (e.g., an automatic mobile robot with a head mounted camera) is allowed to be changed, more detailed information will be collected to improve the performance of recognition.

The idea described above fits into the realm of active object recognition (AOR) [3,4,5], which gathers additional evidence to improve recognition performance by purposefully adjusting the viewpoint (position and orientation) of an agent. Many classic and latest AOR approaches are reviewed in [6,7]. The main focus of AOR research is viewpoint planning (VP) which means how to determine the next best viewpoint of the agent. A good VP policy can greatly ameliorate the recognition performance. In recent years, reinforcement learning has attracted growing research attention on viewpoint planning [8,9,10,11,12]. The agent is able to learn a good VP policy under the guidance of hand-designed reward functions. The main algorithms involved in the learning process are dynamic programming [8] and Q-Learning [9,10,11,12]. Both dynamic programming based and Q-Learning based methods have made a great contribution to AOR. However, these VP methods explore discrete viewpoint space, which have to sample viewpoint space and may bring in significant quantization error.

**Figure 1:**
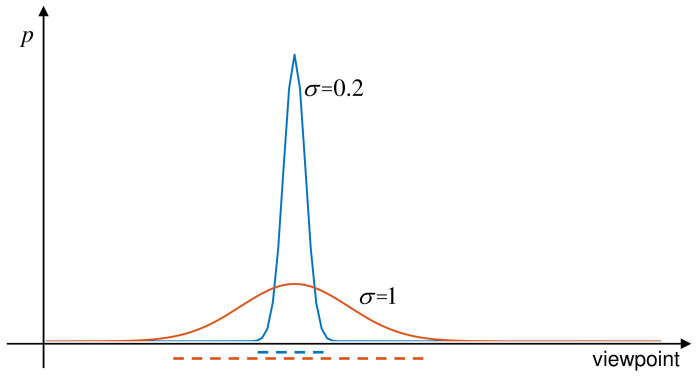
The illustration of viewpoint exploration ability. The exploration ability of the VP policy with the standard deviation σ=1 is stronger than that of the VP policy with the standard deviation σ=0.2. Because there are more possibilities to try new viewpoints when σ=1.

To alleviate this problem, we propose a continuous viewpoint planning approach for AOR based on reinforcement learning in this work. The approach can effectively explore the continuous viewpoint space. To be specific, we employ recently presented proximal policy optimization (PPO) [13] framework to tackle the VP problem. The VP policy is represented by a Gaussian model that can be monotonically improved by the clipping mechanism of PPO. In addition, the standard deviation of the Gaussian model implies the viewpoint exploration ability, which represents the opportunity to try new viewpoints. As shown in Figure 1, the larger the standard deviation is, the stronger the exploration ability is. If the standard deviation is fixed in the whole policy learning process (fixed exploration), two unpleasant results will be produced: (1) the VP policy may stuck in local optimum due to insufficient exploration when the standard deviation is small; (2) the optimal VP policy can not be obtained when the standard deviation is large (because the optimal VP policy is a deterministic policy which is approximately equivalent to a Gaussian model with the small standard deviation). So, in the field of reinforcement learning, it generally hopes to have a higher exploration in the early stage of policy learning and gradually reduce it in the later in order to obtain a better policy [14]. Therefore, we develop a dynamic exploration scheme to automatically adjust viewpoint exploration in the learning process. The scheme is implemented by using separate neural networks for the representation of policy mean and standard deviation and training the mean and standard deviation at the same time. Moreover, entropy regularization [15] is introduced and improved to an adaptive version to prevent the exploration from shrinking prematurely. The experimental results on the public dataset GERMS [12] strongly support the effectiveness of our proposed VP method.

The contributions of our work are as follows:

- A novel continuous viewpoint planning method for active object recognition based on proximal policy optimization is proposed to deal with the problem of quantization error of discrete viewpoint planning methods;
- An adaptive entropy regularization based dynamic exploration scheme is presented to automatically adjust viewpoint exploration in the learning process;
- Experiments are carried out on the public dataset GERMS, and the proposed method obtains rather promising results.

The remainder of the paper is laid out as follows. Section 2 reviews the related research. Section 3 formulates the problem. Section 4 details our continuous viewpoint planning method. Section 5 shows the experiment results and analysis whereas we draw conclusions in Section 6.

## 2. Related Work

This section reviews related work about active object recognition and proximal policy optimization.

**Active Object Recognition:** Becerra et al. [8] model object detection as a Partially Observable Markov Decision Process problem, which is solved using Stochastic Dynamic Programming. In [9], researchers formally define the viewpoint selection as an optimization problem and use reinforcement learning for viewpoint training without user interaction. Malmir et al. [12] contribute a image-based AOR publicly dataset named GERMS and propose a deep Q-learning (DQL) system that learns to actively examine objects by minimizing overall classification error using standard back-propagation and Q-learning. Similarly, Liu et al. develop a hierarchical local-receptive-field-based extreme learning machine architecture to learn the state representation and utilize Q-learning to find the optimal policy [10]. In [11], researchers treat AOR as a Partially Observable Markov Decision Process and find corresponding action-values of training data using belief tree search. All above methods explore discrete viewpoint space, which may miss a few important object information owing to the quantization error of viewpoint. Therefore, we develop a continuous VP method for AOR to address this problem. The closest method to ours in this respect is [16] which resorts trust region policy optimization (TRPO) framework [17] to tackle the quantization error problem and shows better results on the dataset GERMS compared to the Q-Learning methods. However, in the TRPO-based AOR method, linear approximation of the optimization objective and quadratic approximation of the constraint are used to jointly direct policy update, leading to relatively high computation complexity. Although the researchers wisely employ extreme learning machine [18] to alleviate this problem, the learning speed is still unsatisfactory. Different from [16], we adopt a first-order optimization framework PPO [13] for continuous VP learning. It is computationally efficient and is able to guarantee monotonic performance improvement of VP policy. In addition, the VP policy standard deviation in [16] is fixed and small, which makes the viewpoint exploration insufficient during the learning process, resulting in the policy stuck in local optimum. However, we develop a dynamic exploration scheme in our work to automatically adjust the standard deviation in the learning process in order to obtain a better policy.

**Proximal Policy Optimization:** PPO has achieved significant successes in enormous applications. Gangapurwala et al. [19] introduce a guided constrained policy optimization framework based on PPO which guarantees the behavior of real quadruped robot within required safety constraints during training process. A centralized coordination scheme of automated vehicles at an intersection without traffic light using PPO is proposed to solve low computation efficiency suffered by state-of-the-art methods [20]. In [21], researchers apply PPO to the task of image captioning to establish a further improvement for the training phase of reinforcement learning. In [22], researchers propose an integrated metro service scheduling and train unit deployment with a PPO approach based on the deep reinforcement learning framework. A variant of PPO algorithm called memory proximal policy optimization is presented to solve quantum control tasks [23]. In [24], a PPO-based machine learning algorithm is implemented to decide on the replenishments of a group of collaborating companies. However, to our best knowledge, PPO has never been resorted for AOR task. In our work, it is firstly utilized for AOR to learn a continuous VP policy.

## 3. Problem Statement

In a visual AOR system, an agent will be automatically moved to capture images from different viewpoints to recognize an object. The current viewpoint is known to the agent in the recognition system. Specifically, at initial time t=0, the viewpoint of agent is $\varphi_{0}$ and the captured image is $I_{\varphi_{0}}$. According to $I_{\varphi_{0}}$, we can predict the label of the object to be recognized using a classifier. It is often that the single viewpoint image may be not sufficient to give a robust recognition result, we should move the agent to capture more images to improve the recognition performance. This requires us to plan an relative movement action $a_{t}$ (i.e., VP) for the agent to obtain a new viewpoint that is $\varphi_{t\;+\;1}=\varphi_{t}+a_{t}$. Then, the new image $I_{\varphi_{t\;+\;1}}$ captured in the viewpoint $\varphi_{t\;+\;1}$ will be used for the recognition again. The process like this will be repeated until a stop criteria is reached, such as the maximum of *T* steps.

An arbitrary action may lead to a worse view where the captured image does not provide useful information for recognition. Therefore, an effective VP policy is desirable. To this end, we consider the VP problem as a reinforcement learning one which is formulated as a six-element tuple <S,A,r,P,γ,π>. *S* denotes the state space where every element *s* is generated by the images acquired from different viewpoints of an agent. *A* is the continuous action space where every action *a* is used to move the agent to a new viewpoint. r:S×A→R is a reward function designed to assess the value of one action in a certain state. P:S×A×S→[0,1] means the transition probability to the next state when an action is selected in the current state. γ∈[0,1] is a discount factor that represents the difference in importance between future rewards and present rewards. π:S×A→[0,1] is an continuous VP policy that describes the probability of selecting one action to produce a new viewpoint in a certain state. In the reinforcement learning setting, the VP problem is transformed to find the optimal policy $\pi^{*}$, which can move the agent to the best recognition viewpoints.

## 4. Proposed Method

**Figure 2:**
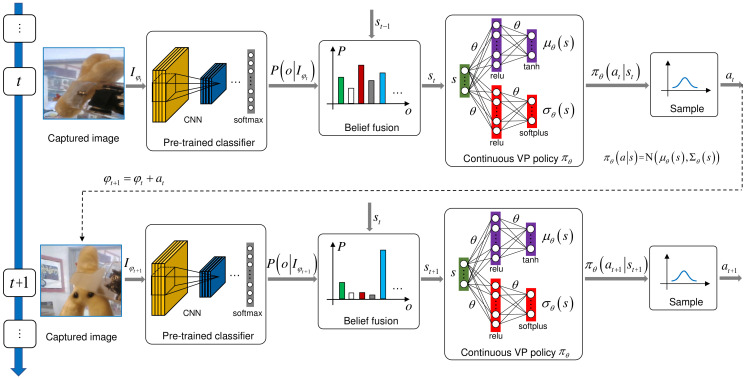
The proposed AOR pipeline. The pipeline adopts PPO framework [13] to learn the continuous VP policy $\pi_{\theta }$ that is denoted by a parameterized Gaussian model. In order to realize dynamic exploration, two separate neural networks are used for the representation of the policy mean and standard deviation of the Gaussian model and trained concurrently. During the training process, the policy $\pi_{\theta }$ is improved by collecting some sample trajectories ${\left\{s_{t},a_{t},r\left(s_{t},a_{t}\right)\right\}}_{t\;=\;0}^{T}$ and optimizing the PPO objective.

To obtain the optimal continuous VP policy $\pi^{*}$ for AOR, we employ PPO framework [13] to tackle this problem. Figure 2 shows our AOR pipeline based on PPO.

During policy training process, at each time step *t*, an agent observes the state $s_{t}\;\in \;S$, takes an action $a_{t}\;\in \;A$ under current VP policy π (i.e., $a_{t}∼\pi \left(a_{t}|s_{t}\right)$), generates a new state $s_{t+1}∼P\left(s_{t+1}|s_{t},a_{t}\right)$, and receives a scalar reward $r(s_{t},a_{t})$. Starting from arbitrary initial state $s_{0}$ at time t=0, the cumulative discounted reward function is

(1) η(π)=Eat∼π(at|st)st+1∼P(st+1|st,at)[∑t=0Tγtr(st,at)],

where E[·] denotes the expectation operator. *T* is the maximum number of planning. η(π) is used to evaluate different VP polices. A better VP policy corresponds to a higher value of η(π). We assume that VP policy π is parameterized by θ and denote it as $\pi_{\theta }$. Thus, to find the optimal continuous VP policy $\pi^{*}$ is to find the optimal parameter $\theta^{*}$ that can be solved by

(2) $$\theta^{*}=\underset{\theta }{\arg\max\;}\eta \left(\pi_{\theta }\right).$$

The recent PPO framework [13] is adopted to address the optimization problem (2) in an iterative updating way. Let $\pi_{\theta_{old}}$ be the old policy, $\pi_{\theta }$ be the new policy after the policy update, and κ(θ) be the probability ratio $\kappa \left(\theta \right)=\pi_{\theta }\left(a_{t}|s_{t}\right)/\pi_{\theta_{old}}\left(a_{t}|s_{t}\right)$. In the PPO framework, $\theta^{*}$ in (2) can be achieved by maximizing a clipping surrogate objective (The detailed derivation process from (2) to (3) can refer to [13,17].):

(3) $$\max_{\theta }L\left(\theta \right)=\underset{\pi_{\theta_{old}}}{E}\left[\min\left(\kappa \left(\theta \right)A_{\pi_{\theta_{old}}}\left(s_{t},a_{t}\right),clip\left(\kappa \left(\theta \right),1-\epsilon ,1+\epsilon \right)A_{\pi_{\theta_{old}}}\left(s_{t},a_{t}\right)\right)\right],$$

where ϵ is a hyper-parameter to control the clipping ratio. $A_{\pi_{\theta_{old}}}\left(s_{t},a_{t}\right)$ is advantage function under the old policy $\pi_{\theta_{old}}$, which is detailed in Section 4.4. In the following, we will elaborate the representation of state $s_{t}$, continuous VP policy $\pi_{\theta }$, and reward function $r(s_{t},a_{t})$ in our PPO-based AOR pipeline and develop a training algorithm to solve the optimization problem in (3).

### 4.1. Belief Fusion for State Representation

As shown in Figure 2, the captured image $I_{\varphi_{t}}$ is first transformed into a series of convolutional neural network (CNN) features. We then add a softmax layer on the top of the CNN model to identify the concerned objects. The output of the softmax layer is a vector that means the recognition belief over different objects. We denote the oth element of the belief vector as $P(o|I_{\varphi_{t}})$ where o=1,2,…,M is the object label. Like [25], the belief $P(o|I_{\varphi_{t}})$ is fused with the accumulated belief $P(o|I_{\varphi_{0}},I_{\varphi_{1}},\ldots ,I_{\varphi_{t-1}})$ from previous images using Naive Bayes:

(4) $$P\left(o|I_{\varphi_{0}},I_{\varphi_{1}},\ldots ,I_{\varphi_{t}}\right)=\beta_{t}P\left(o|I_{\varphi_{t}}\right)P\left(o|I_{\varphi_{0}},I_{\varphi_{1}},\ldots ,I_{\varphi_{t-1}}\right).$$

The fusion result $P(o|I_{\varphi_{0}},I_{\varphi_{1}},\ldots ,I_{\varphi_{t}})$ is the new accumulated belief at time step *t*. $\beta_{t}$ is a normalizing coefficient ($\beta_{t}=1/\sum_{o}P\left(o|I_{\varphi_{t}}\right)P\left(o|I_{\varphi_{0}},I_{\varphi_{1}},\ldots ,I_{\varphi_{t-1}}\right)$) that makes $\sum_{o}P\left(o|I_{\varphi_{0}},I_{\varphi_{1}},\ldots ,I_{\varphi_{t}}\right)=1$ hold. In this work, the accumulated belief is used for the representation of the recognition state (i.e., $s_{t}=P\left(o|I_{\varphi_{0}},I_{\varphi_{1}},\ldots ,I_{\varphi_{t}}\right),o=1,2,\ldots ,M$) at each time step. It is worth noting that the parameters of the classifier (composed of the CNN model and the softmax layer) are pre-trained with the images from different viewpoints of different objects and invariable during the training process of continuous VP policy.

### 4.2. Continuous VP Policy Network Combined with Dynamic Exploration

Similar to [16], the continuous VP policy is represented by a parameterized Gaussian distribution. However, ref. [16] only parameterizes the policy mean μ with a neural network, that is, $\pi_{\theta }\left(a|s\right)=N\left(\mu_{\theta }\left(s\right),\sum \right)$ (Viewpoint is composed of orientation and position, so the planning action *a* may be a multi-dimensional vector. Therefore, the Gaussian model may be a multivariate form. It is usually assumed that the variables in *a* are independent of each other, so the covariance matrix ∑ is a diagonal matrix, i.e., $\sum =diag(\sigma_{1}^{2},\sigma_{2}^{2},\ldots ,\sigma_{d}^{2})$. σ is standard deviation and *d* is the dimension of *a*.). The standard deviations in the covariance matrix ∑ are small and invariable in the whole training process. As analyzed in Section 1, the standard deviation implies the viewpoint exploration ability, the fixed small standard deviation may make the VP policy stuck in local optimum due to insufficient exploration. Therefore, an adaptive entropy regularization based dynamic exploration scheme is developed to automatically adjust the standard deviation in the training process in order to obtain a better policy. The research process and implementation details of the scheme are as follows.

**Parameterization of the Policy Mean and Standard Deviation:** The scheme is first realized by concurrently parameterizing the policy mean and standard deviations with two separate neural networks ($\mu_{\theta }\left(s\right)$ or μ(s;θ) and $\sigma_{\theta }\left(s\right)$ or σ(s;θ)) and training them at the same time. As shown in Figure 2, $\mu_{\theta }\left(s\right)$ and $\sigma_{\theta }\left(s\right)$ are two single hidden-layer fully-connected neural networks which take state as input and output the mean vector and standard deviation vector. The parameters of them are collectively called θ. Consequently, the VP policy is recorded as $\pi_{\theta }\left(a|s\right)=N\left(\mu_{\theta }\left(s\right),\sum_{\theta }\left(s\right)\right)$ which is expanded to

(5) $$\pi_{\theta }\left(a|s\right)=\prod_{i=1}^{d}\frac{1}{\sqrt{2\pi }\sigma_{i}\left(s;\theta \right)}\exp^{-\frac{{\left(a_{i}-\mu_{i}\left(s;\theta \right)\right)}^{2}}{2\sigma_{i}{\left(s;\theta \right)}^{2}}}.$$

The *i*th element of the mean vector and standard deviation vector are represented as $\mu_{i}\left(s;\theta \right)$ and $\sigma_{i}\left(s;\theta \right)$, respectively. *d* is the dimension of action *a*. During training, the update of parameter θ under the PPO framework will simultaneously affect the policy mean and standard deviations, leading to the dynamic exploration.

**Figure 3:**
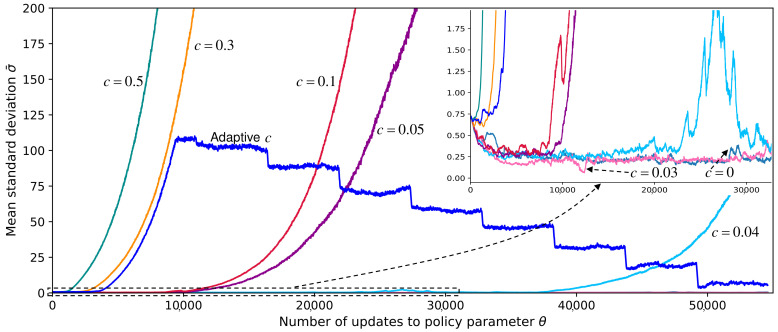
The changes of exploration ability in the training process of GERMS left arm dataset [12] under different dynamic exploration schemes. Because the standard deviation is a function of the state, the standard deviation representing the exploration ability refers to the average of the standard deviation $\bar{\sigma }$ corresponding to all states. However, there are infinite states, so $\bar{\sigma }$ can not be calculated. In the training, we use the average of the standard deviation of some sample states to approximately replace $\bar{\sigma }$. We implement three dynamic exploration schemes step by step: (1) the first is the simultaneous parameterization of policy mean and standard deviation with two separate neural networks (the curve with c=0); (2) the second is to add the constant coefficient entropy regularization on the basis of (1) (the curves with c=0.03,0.04,0.05,0.1,0.3, or 0.5); (3) the third is that the constant coefficient is improved into an adaptive version on the basis of (2) (the curve with Adaptive *c*). After experimental comparison, scheme (3) can meet our dynamic exploration need.

**Entropy Regularization:** As stated in Section 1, in reinforcement learning, it generally hopes to have a higher exploration in the early stage of policy learning and gradually reduce it in the later in order to obtain a better policy [14]. However, we find the standard deviations shrink prematurely and adjust in a small range in the training process. As shown in Figure 3, it is the change of standard deviation in the training process of GERMS dataset [12] which has a single action dimension (A shrinkage case with two action dimensions is shown in [14]). It shrinks rapidly to a small value soon after the beginning of training and always keeps in a small value range (the curve with c=0 in Figure 3), which may also result in the insufficient exploration. To address this problem, we then introduce entropy regularization [15] to the PPO optimization objective (3) to prevent the exploration from shrinking prematurely. Therefore, (3) is transformed into:

(6) $$\begin{array}{c} \begin{array}{cc} \; & \max_{\theta }L^{Ent}\left(\theta \right)=\underset{\pi_{\theta_{old}}}{E}[\min(\kappa \left(\theta \right)A_{\pi_{\theta_{old}}}\left(s_{t},a_{t}\right),\\ & clip\left(\kappa \left(\theta \right),1-\epsilon ,1+\epsilon \right)A_{\pi_{\theta_{old}}}\left(s_{t},a_{t}\right))+cH\left(\pi_{\theta }\left(\cdot |s_{t}\right)\right)], \end{array} \end{array}$$

where *c* is a constant coefficient and H(·) is entropy operator (H(x)=−∫p(x)logp(x) or H(x)=−∑p(x)logp(x). The entropy of a multivariate normal distribution is $\frac{1}{2}\log{\left(2\pi e\right)}^{d}\left|\sum \right|$.).

**Figure 4:**
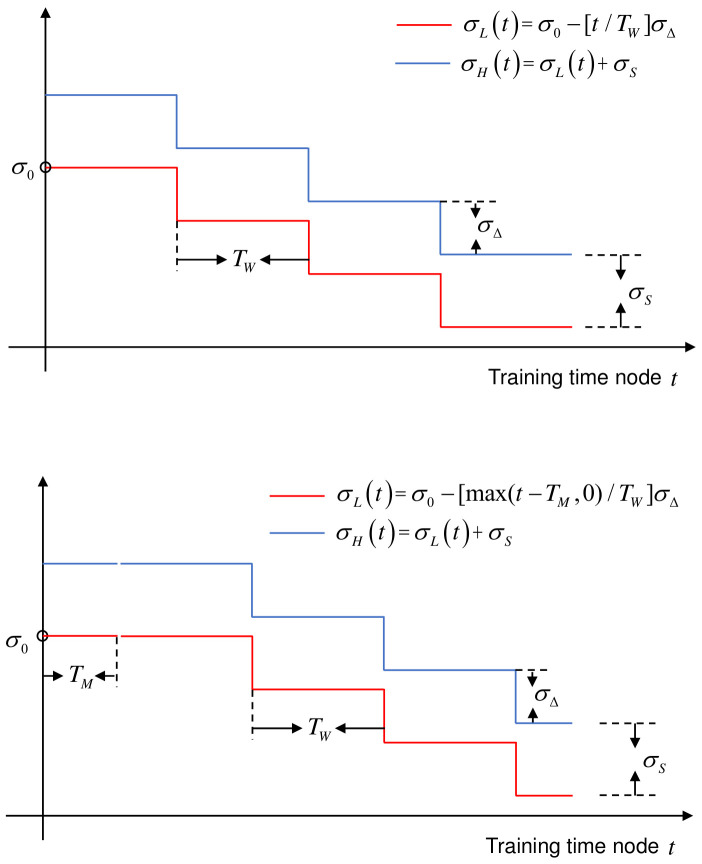
The diagram of upper and lower boundary functions of standard deviation.

**Adaptive Entropy Regularization Coefficient:** In our experiment, we find the constant coefficient *c* in (6) is a hyper-parameter that is difficult to tune. As shown in Figure 3, when *c* is less than or equal to 0.03, entropy regularization fails to prevent the premature decay of exploration; when *c* is greater than 0.03, the standard deviation increases explosively. Thus, to tackle this problem, we last propose an adaptive entropy regularization method that can adapt the coefficient to achieve the appropriate exploration ability in the training process. The coefficient *c* in (6) is improved to

(7) $$c=\left\{\begin{array}{ccc} c_{div}, & \; & \exists i\;{\bar{\sigma }}_{i}<\sigma_{L_{i}}\left(t\right)\\ -c_{div}, & \; & \exists i\;{\bar{\sigma }}_{i}>\sigma_{H_{i}}\left(t\right),\\ 0, & \; & otherwise \end{array}\right.$$

where $c_{div}$ is a divergence coefficient such as 0.04, 0.05, 0.1, 0.3, and 0.5 in Figure 3. If the planning action is multidimensional, then $c_{div}$ is a coefficient that makes the standard deviation of each dimension diverge. $\sigma_{H_{i}}\left(t\right)$ and $\sigma_{L_{i}}\left(t\right)$ are the i-dimensional upper and lower boundaries of the standard deviation you want to maintain in the training. They are the functions of training time node *t*. In our work, we model them as stage functions shown in Figure 4. To be specific, the stage functions in a certain dimension are defined as

(8) $$\begin{array}{c} \begin{array}{cc} \; & \sigma_{L}\left(t\right)=\sigma_{0}-\left[max\left(t-T_{M},0\right)/T_{W}\right]\sigma_{\Delta }\\ \; & \sigma_{H}\left(t\right)=\sigma_{L}\left(t\right)+\sigma_{S}. \end{array} \end{array}$$

$T_{W}$ is the training duration of each stage. According to it, the total training time can be evenly divided into several stages. $\sigma_{0}$ is the initial standard deviation. $\sigma_{\Delta }$ is the increment of the standard deviation. $\sigma_{S}$ is the boundary range. [·] is the rounding operator, e.g., [1/3]=0. $max(t-T_{M},0)$ is to increase the training time of the first stage by $T_{M}$. As shown in Figure 3, this is because it takes some time to raise the standard deviation to the boundary value of the first stage at the beginning of training.

After experimental verification, the dynamic exploration with adaptive entropy regularization can meet our exploration requirement.

### 4.3. Reward Setting

Reward function $r(s_{t},a_{t})$ plays an important role in encouraging effective viewpoint selection. In Section 4.1, the recognition state ($s_{t}=P\left(o|I_{\varphi_{0}},I_{\varphi_{1}},\ldots ,I_{\varphi_{t}}\right),o=1,2,\ldots ,M$) describes a probability distribution over different objects. The flatter the distribution is, the stronger the recognition ambiguity is. Here, we resort information entropy [26,27] to quantify the ambiguity. Then the ambiguity in state $s_{t}$ is represented as $H(s_{t})$. The goal of AOR is to eliminate this ambiguity to improve recognition performance by viewpoint planning. A beneficial viewpoint attempt can reduce the current ambiguity. Therefore, we design the reward function according to the ambiguity in different states after viewpoint selection. Let ${\hat{o}}_{t+1}$ be the predicted result and $o^{*}$ be the label of the image in the new viewpoint ($I_{\varphi_{t+1}}=I_{\varphi_{t}}+a_{t}$). Among them, ${\hat{o}}_{t\;+\;1}=argmax_{o}P\left(o|I_{\varphi_{0}},I_{\varphi_{1}},\ldots ,I_{\varphi_{t\;+\;1}}\right)$. If the predicted result ${\hat{o}}_{t\;+\;1}$ is right and the information entropy $H(s_{t\;+\;1})$ is smaller than $H(s_{t})$ in state $s_{t}$, it means that the VP action $a_{t}$ in state $s_{t}$ is useful for recognition. Then the agent will receive a positive reward. Otherwise, the reward is non positive when the entropy does not decrease or the prediction is wrong. To sum up, the reward function is formulated as

(9) $$r\left(s_{t},a_{t}\right)=\left\{\begin{array}{ccc} -1, & \; & {\hat{o}}_{t\;+\;1}\neq o^{*}\\ 0, & \; & {\hat{o}}_{t\;+\;1}=o^{*},H\left(s_{t\;+\;1}\right)\geq H\left(s_{t}\right),\\ 1, & \; & {\hat{o}}_{t\;+\;1}=o^{*},H\left(s_{t\;+\;1}\right)<H\left(s_{t}\right) \end{array}\right.$$

where $r(s_{t},a_{t})$ can be denoted as $r_{t}$ for simplicity.

### 4.4. Training the Policy Network

To solve the optimization problem in (6), we develop a training algorithm to iteratively update θ in the policy network. The algorithm shown in Algorithm 1 is Actor–Critic style [15].

To replace the expectation operator in (6), we apply Monte Carlo method [28] to deal with it in an approximate manner. Specifically, we repeat *N* times to run the old policy $\pi_{\theta_{old}}$ for *T* time steps to collect a trajectory ${\left\{s_{t},a_{t},r_{t},s_{t\;+\;1}\right\}}_{t=0}^{T}$. With *N* trajectories, (6) can be approximated as:

(10) $$\begin{array}{c} \begin{array}{cc} \; & \max_{\theta }{\hat{L}}^{Ent}\left(\theta \right)=\frac{1}{N(T\;+\;1)}\sum_{i\;=\;1}^{N}\sum_{t\;=\;0}^{T}[\min(\kappa^{\left(i\right)}\left(\theta \right)A_{\pi_{\theta_{old}}}^{\left(i\right)}\left(s_{t},a_{t}\right)\\ & ,clip\left(\kappa^{\left(i\right)}\left(\theta \right),1-\epsilon ,1+\epsilon \right)A_{\pi_{\theta_{old}}}^{\left(i\right)}\left(s_{t},a_{t}\right))+cH\left(\pi_{\theta }^{\left(i\right)}\left(\cdot |s_{t}\right)\right)]. \end{array} \end{array}$$

The advantage function $A_{\pi_{\theta_{old}}}\left(s_{t},a_{t}\right)$ can be estimated using the technology of generalized advantage estimation (GAE) [29]:

(11) $$\begin{array}{c} \begin{array}{cc} \; & A_{\pi_{\theta_{old}}}\left(s_{t},a_{t}\right)=\delta_{t}+\left(\gamma \lambda \right)\delta_{t\;+\;1}+\ldots +{\left(\gamma \lambda \right)}^{T-t}\delta_{T},\\ \; & where\;\delta_{t}=r_{t}+\gamma V_{\pi_{\theta_{old}}}\left(s_{t\;+\;1}\right)-V_{\pi_{\theta_{old}}}\left(s_{t}\right). \end{array} \end{array}$$

$V_{\pi_{\theta_{old}}}\left(\cdot \right)$ is state value function under the old VP policy $\pi_{\theta_{old}}$. It is approximately represented by a two-layer fully connected network with parameter ω. The network maps the state $s_{t}$ to the function value $V(s_{t};\omega )$. We update ω to obtain the state value function corresponding to different VP policies. We use the *N* trajectories (sampled by $\pi_{\theta_{old}}$) again to fit the state value function $V_{\pi_{\theta_{old}}}\left(s_{t};\omega \right)$ of the old policy $\pi_{\theta_{old}}$ by solving the optimization problem:

(12) $$\min_{\omega }\hat{L}\left(\omega \right)=\frac{1}{N(T\;+\;1)}\sum_{i\;=\;1}^{N}\sum_{t\;=\;0}^{T}{\left(V_{Target}^{\left(i\right)}\left(s_{t}\right)-V_{\pi_{\theta_{old}}}^{\left(i\right)}\left(s_{t};\omega \right)\right)}^{2}.$$

**Algorithm 1:** Training the continuous VP policy network

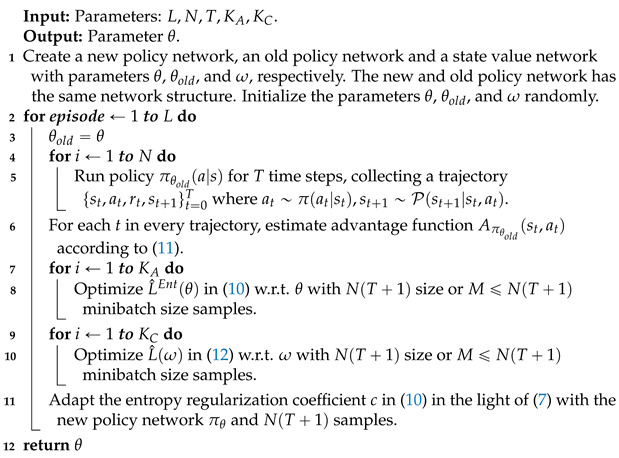



$V_{Target}\left(s_{t}\right)$ is not involved in the optimization procedure. It is calculated using $V_{Target}\left(s_{t}\right)=r_{t}+\gamma r_{t+1}+\ldots +\gamma^{T-t-1}r_{T-1}+\gamma^{T-t}V_{\pi_{\theta_{old}}}\left(s_{T};\omega \right)$ in advance.

The iterative update process of (10) and (12) is shown in lines 7–10 of Algorithm 1.

Once the optimal parameter $\theta^{*}$ is obtained, it can be used for the practical AOR task. In state $s_{t}$, the planned action is $a_{t}∼N\left(\mu_{\theta^{*}}\left(s_{t}\right),\sum_{\theta^{*}}\left(s_{t}\right)\right)$, and the next best viewpoint is $\varphi_{t+1}=\varphi_{t}+a_{t}$.

## 5. Experiments

### 5.1. Experimental Setup

**Figure 5:**
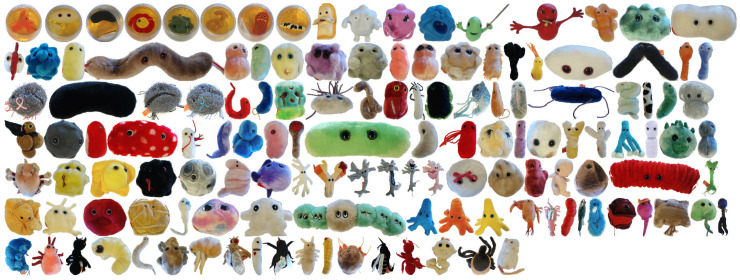
The GERMS dataset [12]. The objects are soft toys describing various human cell types, microbes and disease-related organisms.

**Figure 6:**
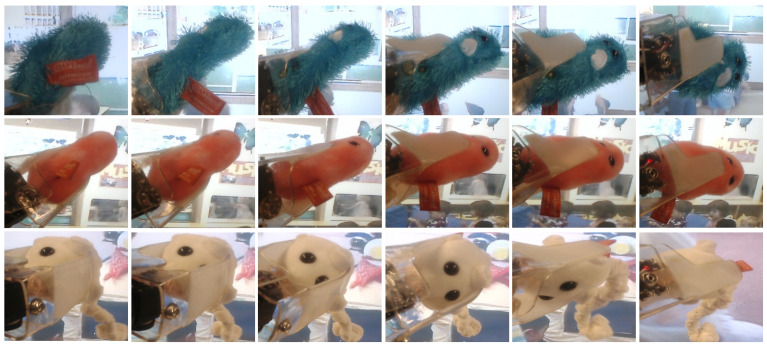
The images from different viewpoints in different tracks.

**Dataset and Metric:** The GERMS dataset [12] shown in Figure 5 is collected in the context of the RUBI project whose intention is to develop a robot that interact with toddlers in early childhood education. It is composed of 1365 video tracks of give-and-take trials using 136 different soft toy objects. The tracks are divided according to the arm of the robot, with roughly half the training and testing tracks being the left arm and the other half the right arm. Each trial generates a track that records the robot putting the grasped object in its center of view, rotating it by 180° and then returning it. During the trial, the robot continuously saves images from its head-mounted camera at 30 frames per second, as shown in Figure 6. Meanwhile, the joint position and object label are recorded. These data are stored in a track, a series of which constitutes the dataset. On average, each track contains 150 images, Table 1 outlines the number of images in the dataset. These joint positions in each track allow researchers to simulate different VP methods in one dimensional action space. The performance of different VP methods is evaluated using recognition accuracy that is the average of the entire test set.

**Implementation Details:** In this work, we employ the Tensorflow platform [30] to implement the proposed method. The CNN model used in the pre-trained classifier is VGG-net provided in [12], which can transform each image in GERMS into a 4096-dimensional feature vector. The number of neurons in the last softmax layer of the pre-trained classifier is 136. In the policy $\mu_{\theta }\left(s\right)$ network, the number of neurons and the activation function in the hidden layer are 1024 and relu; The last layer uses tanh activation function and has one neuron. In order to match the viewpoint range of GERMS, we multiply the output of tanh by 512, so that the next relative VP action range is [−45°, 45°]. In the policy $\sigma_{\theta }\left(s\right)$ network, the configuration of the hidden layer is consistent with that in $\mu_{\theta }\left(s\right)$; The number of neurons and the activation function in the last layer are 1 and softplus. The configuration of the hidden layer in the state value network $V_{\omega }\left(s\right)$ is same as that in $\mu_{\theta }\left(s\right)$. The reward discount factor γ is 0.96, and the GAE parameter λ is 0.95. The clipping ration parameter ϵ is empirically set as ϵ=0.2 in the light of the original implementation of PPO [13]. The VP policy converges after 4200 episodes in the training process, therefore, we set L=4200. *N* and the minibatch size *M* are all 128. $K_{A}$ and $K_{C}$ are 1 and 10. The maximum step *T* for recognition is set as T=12. The Adam optimizer [31] is used for the optimization of the policy network and the state value network. The learning rates of them are 0.0001 and 0.0002. In the dynamic exploration, the parameters $c_{div}$, $\sigma_{0}$, $T_{M}$, $T_{W}$, $\sigma_{\Delta }$ and $\sigma_{S}$ are 0.3, 106, 3, 3, 14, and 14, respectively.

### 5.2. Ablation Study

To investigate the effectiveness of our dynamic exploration scheme, we intend to conduct the variant experiments with different components ablation. Table 2 shows the abbreviations and interpretations of different components. In the variant experiments, the components AERC, ER, and SSDN are gradually removed.

**Figure 7:**
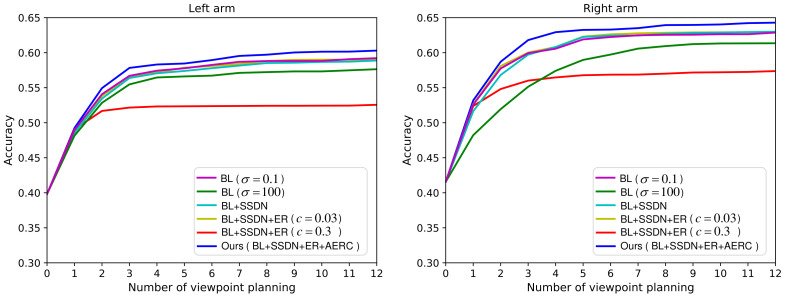
The performance comparison results of ablation experiments.

The experimental results are presented in Figure 7, where the recognition accuracy is a function of the number of planned actions. From Figure 7, we can notice that the performance degrades heavily after removing the component AERC. The results of the experiments BL(σ=0.1), BL+SSDN, and BL+SSDN+ER(c=0.03) are similar. This is because their exploration ability is all at a low level. Although the experiment BL(σ=100) has a high exploration ability, the VP policy can not converge to the optimal. So its result is slightly worse. The result of experiment BL+SSDN+ER(c=0.3) is the most unsatisfactory, because its standard deviation increases explosively as shown in Figure 3. This study validates the effectiveness of our proposed adaptive entropy regularization based dynamic exploration scheme.

### 5.3. Dynamic Exploration Study

**Figure 8:**
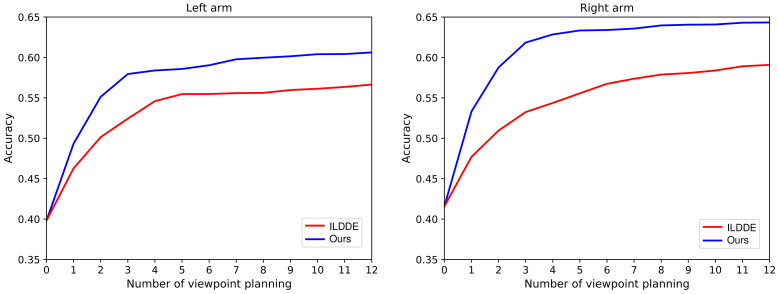
The performance comparison results of continuous VP policies combined with different dynamic exploration schemes. The parameters $\sigma_{0}$, $\sigma_{L}$, and $T_{L}$ involved in ILDDE are 120, 0.1, and 4200.

In our dynamic exploration scheme, the standard deviation σ is adapted by updating the VP policy parameters θ during the training. Another natural idea (i.e., independent linear decaying dynamic exploration, ILDDE) is to adjust σ independently of parameters θ. The idea is realized as

(13) $$\sigma \left(t\right)=\frac{\left(\sigma_{L}-\sigma_{0}\right)}{T_{L}}t+\sigma_{0}\;\;\;\;\;\left(\sigma_{L}<\sigma_{0}\right),$$

where σ is a linear decaying function of the training time node *t*. $\sigma_{0}$ and $\sigma_{L}$ are the initial and final σ values, respectively. $T_{L}$ is the total training time. Therefore, the VP policy can be represented as $\pi_{\theta }\left(a|s\right)=N\left(\mu_{\theta }\left(s\right),\sum \left(t\right)\right)$ where $\sum \left(t\right)=diag(\sigma_{1}^{2}\left(t\right),\sigma_{2}^{2}\left(t\right),...,\sigma_{d}^{2}\left(t\right))$. In the training, the update of parameters θ only affects the policy mean, the policy standard deviation is independently adapted by (13). We experiment with this idea and compare it with our scheme. In the experiment, except that the independent network $\sigma_{\theta }\left(s\right)$ in Figure 2 is removed and replaced with σ(t) in (13), everything else is exactly the same. From the presented results in Figure 8, we can notice that the performance of our scheme is much better than that of ILDDE. This is because the VP policy corresponding to ILDDE is affected by two parameters: θ and *t*. However, *t* does not participate in the optimization process, which may make the learned policy worse and worse. However, in our scheme, the policy mean and standard deviation are only related to θ, and participate in the whole optimization process.

### 5.4. Comparison with the State-of-the-Art Methods

In this subsection, several baselines [10] and state-of-the-art VP approaches [11,12,16] are employed for experiment comparison with our continuous VP method, which are showed as follows:

- Random policy [10] plans a random action from the action space $\left\{\pm \frac{\pi }{64},\pm \frac{\pi }{32},\pm \frac{\pi }{16},\pm \frac{\pi }{8},\pm \frac{\pi }{4}\right\}$ with uniform probability;
- Sequential policy [10] moves the agent to the next immediate position in the same direction;
- DQL policy [11,12] exploits deep Q-Learning algorithm to learn a discrete VP policy. The discrete action space is $\left\{\pm \frac{\pi }{64},\pm \frac{\pi }{32},\pm \frac{\pi }{16},\pm \frac{\pi }{8},\pm \frac{\pi }{4}\right\}$;
- E-TRPO policy [16] develops a continuous VP method which is implemented by trust region policy optimization [17] and extreme learning machine [18]. It represents the VP policy as a Gaussian model and learns the policy with a fixed exploration scheme.

**Figure 9:**
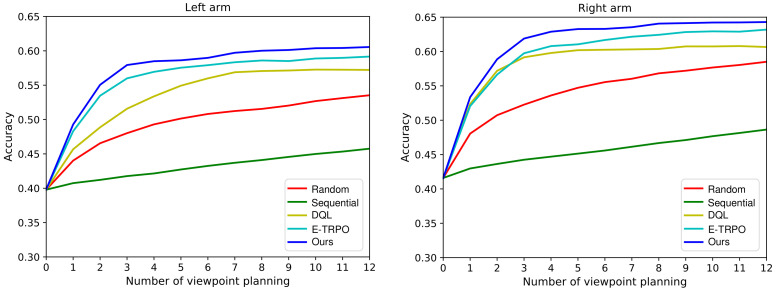
Performance comparison between our proposed continuous VP method and several competing approaches.

For a fair comparison, the classifiers of different methods are the same in the experiment. The evaluation results of our VP model against other approaches are presented in Figure 9, from which we have the following observations: (1) Our proposed method achieve better performance compared with the state-of-the-art methods; (2) The performance of active policy is significantly better than that of passive policy. Random policy and Sequential policy are essentially passive VP policies. They do not actively plan the next viewpoint according to the information obtained from the previous viewpoints. However, DQL policy, E-TRPO policy, and the proposed method use the previous information to plan the next viewpoint, so they are active VP policies; (3) The performance of continuous VP policy outperforms that of discrete VP policy. DQL policy is a discrete VP policy while E-TRPO policy and our method are continuous VP policies. The continuous VP policy explores in the continuous viewpoint space and will not miss some important viewpoints; (4) Compared with the continuous VP method E-TRPO, our continuous VP model has better performance. This is mainly because we present an effective dynamic exploration scheme, which can explore more new viewpoints and find better solutions.

## 6. Conclusions

In this work, we develop a continuous viewpoint planning method for active object recognition based on reinforcement learning. More specifically, the viewpoint planning policy is represented as a parameterized Gaussian model and learned using the proximal policy framework. We also design a dynamic exploration scheme based on adaptive entropy regularization to automatically adjust the viewpoint exploration ability in the learning process. Experiments on the public dataset GERMS show the superiority of our method.

## Figures and Tables

**Table 1:** GERMS dataset statistics (mean ± std).

	Number of Tracks	Images/Track	Total Number of Images
Train	816	157 ± 12	76,722
Test	549	145 ± 19	51,561

**Table 2:** Abbreviations and interpretations for different components in our dynamic exploration scheme.

Abbreviation	Interpretation
BL	Baseline PPO framework [13] with a fixed exploration scheme (i.e., the standard deviation σ is a constant)
SSDN	Separate standard deviation network
ER	Entropy regularization (with a fixed coefficient)
AERC	Adaptive entropy regularization coefficient

## Data Availability

The dataset used in this work are available at https://sites.google.com/a/eng.ucsd.edu/mmalmir/code-software-datasets, accessed on 1 November 2021.
